# Effects of Race, Ethnicity and Socioeconomic Deprivation on Postpartum Haemorrhage in High‐Income Countries: A Systematic Review and Meta‐Analysis

**DOI:** 10.1111/1471-0528.18278

**Published:** 2025-07-09

**Authors:** Amy Elsmore, Gbenga Alayande, Elizabeth Mainwaring, Mahfam Jafarpour, Michael P. Rimmer, Neil Cockburn, Jason Curtis, Ragave Ilaalagan, Bassel Al‐Wattar, Sarah Bell, Bala Karunakaran, William Parry‐Smith, Pensee Wu

**Affiliations:** ^1^ Department of Obstetrics and Gynaecology The Shrewsbury and Telford NHS Trust Shrewsbury UK; ^2^ School of Medicine Keele University Keele Staffordshire UK; ^3^ Department of Obstetrics and Gynaecology The Royal Wolverhampton NHS Trust Wolverhampton UK; ^4^ School of Medicine The University of Cambridge Cambridge UK; ^5^ The Centre for Reproductive Health Institute of Regeneration and Repair, University of Edinburgh Edinburgh UK; ^6^ Edinburgh Fertility Centre, Simpsons Centre for Reproductive Health NHS Lothian Edinburgh UK; ^7^ Department of Applied Health Science University of Birmingham Birmingham UK; ^8^ Shrewsbury and Telford Health Libraries The Shrewsbury and Telford NHS Trust Shrewsbury UK; ^9^ Clinical Trials Unit Faculty of Health Medicine and Social Care, Anglia Ruskin University Chelmsford UK; ^10^ Beginnings Assisted Conception Unit Epsom and St Helier University Hospitals London UK; ^11^ Department of Anaesthesia Cardiff and Vale University Health Board Cardiff UK; ^12^ Department of Obstetrics and Gynaecology College of Medicine, National Cheng Kung University Tainan Taiwan; ^13^ Academic Department of Obstetrics and Gynaecology University Hospital North Midlands Stoke‐on‐Trent UK

**Keywords:** ethnicity, maternal health disparities, postpartum haemorrhage, race, socioeconomic deprivation

## Abstract

**Background:**

Postpartum haemorrhage (PPH) is a leading cause of mortality and morbidity globally. While individual studies have revealed disparities in outcomes, a comprehensive summary of PPH risk across diverse groups is lacking.

**Objectives:**

To quantify the association between ethnicity, deprivation and risk of PPH in high‐income countries (HICs).

**Search Strategy:**

A systematic search of MEDLINE, CINAHL, EMBASE and Google Scholar from inception to 20 August 2024.

**Selection Criteria:**

Observational and experimental studies from HICs that reported the outcome of PPH in at least two ethnic or socioeconomic groups.

**Data Collection and Analysis:**

Two reviewers performed independent data extraction. A random‐effects model was used to estimate the risk. A subgroup analysis was performed by geographical region and time period.

**Main Results:**

A total of 79 studies with 169 579 388 women were included, spanning 15 HICs. Ethnic minority women experienced a higher risk of PPH compared to the majority White or European group. This was seen across Black (OR 1.16, 95% CI 1.09, 1.23), Asian (OR 1.33, 95% CI 1.27, 1.39), Hispanic (OR 1.20, 95% CI 1.12, 1.29) and women from the minority ethnic group within a given study (OR 1.13, 95% CI 1.03,1.24). Due to data limitations, eight studies on PPH and socioeconomic status were summarised narratively, indicating a higher PPH risk for those experiencing deprivation.

**Conclusions:**

Women from an ethnic minority background or exposed to socioeconomic deprivation had an increased risk of PPH in HICs. Standardisation of data collection for ethnicity and socioeconomic status is recommended to accurately quantify and address these disparities.

## 1 Introduction

Postpartum haemorrhage (PPH), the primary cause of maternal death globally [1], exhibits increasing incidence linked to factors such as rising maternal obesity, advanced age and caesarean section rates [2]. Despite well‐resourced healthcare in high‐income countries (HICs), significant disparities exist in maternal and foetal outcomes. Ethnic and racial disparities have been observed in the United Kingdom (UK) maternal mortality reports since ethnicity data collection started in 2000 [3]. Women from Black and South Asian backgrounds are more likely to experience complications such as PPH, even after adjusting for clinical factors [4], a trend echoed in the United States of America (USA) [5, 6]. Socioeconomic deprivation is also linked to adverse pregnancy outcomes; in the UK, women living in the most deprived areas are twice as likely to die as those from the least deprived areas [7]. There is an overlap between minority ethnic groups and socioeconomic deprivation [3, 7]. These factors may interact to compound adverse outcomes through complex interactions involving healthcare access, risk underestimation, and systemic bias [3].

While existing research on these disparities in HICs often focuses on individual studies [4, 6], comprehensive reviews are limited. Sheikh et al. [8] highlighted increased perinatal risks for Black and Hispanic women, but not specifically PPH. Okunlola et al. [9] identified higher PPH risk in Hispanic, Asian, and Native Hawaiian and Other Pacific Islander (NHOPI) women, and a greater rate of blood transfusion for White women, but lacked a meta‐analysis. Evidence specifically and comprehensively reviewing the association between socioeconomic status and PPH remains limited.

Measuring the association between race, ethnicity, socioeconomic deprivation, and PPH is fundamental to addressing maternal health disparities for this outcome. This comprehensive review and meta‐analysis aim to identify and determine the extent of these risks, providing evidence to inform targeted interventions and policies aimed at achieving equity in maternal outcomes. Understanding the scope of these potential disparities is a necessary first step in strategising how to reduce unacceptable inequalities in PPH incidence and its consequences. The findings may inform the development of culturally sensitive interventions, such as the prioritisation of resources for at‐risk communities and the implementation of policies aimed at mitigating systemic biases within healthcare delivery to ensure equitable care for all women.

## 2 Methods

A systematic review and meta‐analysis protocol was developed following the Preferred Reporting Items for Systematic Reviews and Meta‐Analysis Protocols (PRISMA‐P) guidelines [10] that was registered with PROSPERO (CRD42023457160) [11]; no amendments have been made to the protocol after registration.

### 2.1 Search Strategy and Selection Criteria

Searches of MEDLINE, EMBASE, CINAHL, and Google Scholar were performed from inception to 20 August 2024 with the assistance of a librarian (JC). Bibliography searching of included studies was also performed to identify relevant literature. The detailed search strategy and search terms are outlined in the (Appendix S1). Studies were restricted to human studies, and no language restrictions were applied.

Observational and experimental studies that reported PPH by maternal ethnicity or socioeconomic status as the exposure of interest were selected. Included studies had at least two ethnic or racial groups (one majority group, most commonly White, as the comparator, and at least one minority group), or at least two socioeconomic groups, and reported sufficient data to allow pooled effect estimates to be calculated. Studies conducted in HICs were selected, as per the World Bank classification [12]. Studies reporting low‐ and middle‐income settings were excluded.

All titles and abstracts were independently screened by two reviewers (a combination of AE, GA, MJ, LM and RI) using Covidence systematic review software (Veritas Health Innovation, Melbourne, Australia). This was followed by independent full‐text screening by AE, EM, MJ and GA. Independent double data extraction was performed by two reviewers (AE and MR). Discrepancies were resolved through consensus with a third reviewer if required. Data were collected on study design, year, country, number of participants, definition of PPH and number of PPH events and outcomes, ethnicities and measures of socioeconomic status described in the study. Studies obtained data on ethnicity and socioeconomic status through various methods, including self‐report, routine data collected in medical records, or recorded by the research team. The information was obtained from published data.

Outcomes investigated included PPH, severe maternal morbidity (SMM) due to haemorrhage, intensive care admission, blood transfusion and maternal death due to haemorrhage.

### 2.2 Risk of Bias Assessment

Study quality was assessed against the Newcastle‐Ottawa Quality Assessment Scale (NOS) for cohort studies and case‐control studies [13]. For cross‐sectional studies included in the review, this scale was adapted, as has been demonstrated in other reviews [14] (see Appendix S2). The risk of bias was independently assessed by four reviewers (AE, GA, MJ and MR), with conflicts resolved through discussion.

### 2.3 Data Analysis

Studies that were pooled were assessed by reviewers to be sufficiently clinically homogeneous. Acknowledging the heterogeneous reporting of racial and ethnic data in the primary studies, these categories were combined under the broader term ‘ethnicity’ for the purpose of this meta‐analysis. Review Manager 5.4 (RevMan version 5.4, The Cochrane Collaboration) was used to conduct the meta‐analysis, using a random‐effects model, using the inverse variance method to weight the studies. This was chosen because the included studies were conducted in a wide range of settings in different populations, with heterogeneity in study design, definition of PPH and methods categorising ethnicity and socioeconomic status. The *I* ^2^ statistic assessed statistical heterogeneity. Publication bias was assessed by the symmetry of funnel plots (in outcomes that included more than ten publications). Pooled effect estimates were calculated using odds ratios (ORs) and 95% confidence intervals (CIs) for dichotomous outcomes that is different ethnicities (Black, Hispanic and Asian) with White women serving as the reference group. For studies where this was not feasible due to the description of ethnicity, the pooled effect estimate was calculated for the majority ethnicity reported in the study versus the minority ethnicity. For studies reporting maternal country or continent of origin, ethnicity was inferred based on geographical region to allow for broader data inclusion. To be comprehensive, the definition of PPH or SMM was not specifically defined at the outset and was included in the data extraction. Despite the heterogeneity in PPH definitions across included studies, the decision to pool data was made to synthesise the overall evidence of the association with ethnicity and deprivation. Leave‐one‐out analysis was performed to explore the effect of any one study that influenced the effect size or contributed to heterogeneity.

Subgroup analysis was performed to explore heterogeneity, to investigate the impact of study continent on the risk of PPH (North America, Europe, Australia/New Zealand and Other), and time period (studies conducted 1990–1999, 2000–2009 and 2010–2019), to explore potential effects related to healthcare practices and population characteristics that might vary across these contexts. All studies were classified based on whether the start dates of the study were within the specified time period.

To assess if any identified associations were independent of established PPH risk factors, we performed a sensitivity analysis on adjusted estimates for clinical confounders. These included maternal age, body mass index (BMI), parity, maternal medical co‐morbidities (including hypertension and diabetes), induction or augmentation of labour, mode of birth and foetal macrosomia, and were chosen based on established evidence from national guidance [15] and comprehensive reviews [16] and were commonly adjusted for in the included studies.

For studies whose data were not amenable to meta‐analysis, a narrative approach was used to summarise the findings. All studies reporting the incidence of PPH by socioeconomic group or status were analysed via narrative summary.

### 2.4 Core Outcome Sets and Patient Involvement

A PPH core outcome [17] set was considered when selecting study outcomes. No patient involvement or direct patient contact occurred in this study.

## 3 Results

### 3.1 Characteristics of Included Studies

**FIGURE 1:**
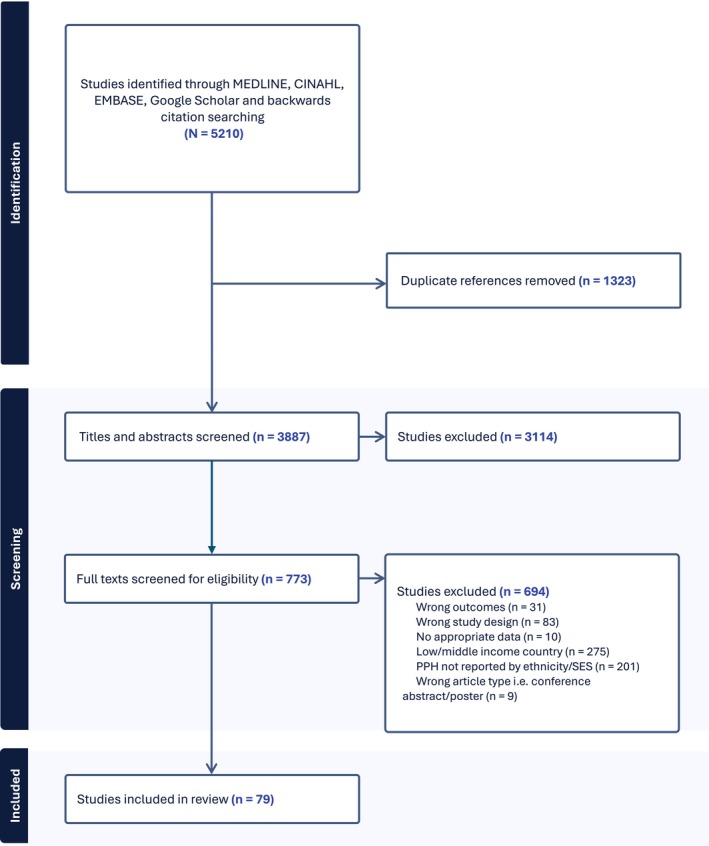
Flow chart of study selection in the systematic review of associations between ethnicity, socioeconomic status, and postpartum haemorrhage.

The initial searches produced 5210 references; after de‐duplication, 3887 titles and abstracts were screened. Full‐text screening was performed with manual searching of the bibliographies of included studies to identify any further relevant articles. 79 studies were included in this review (Figure 1).

The detailed characteristics of the included studies and study populations are demonstrated in Table S1. We included 66 cohort studies, with 62 retrospective [4, 6, 18, 19, 20, 21, 22, 23, 24, 25, 26, 27, 28, 29, 30, 31, 32, 33, 34, 35, 36, 37, 38, 39, 40, 41, 42, 43, 44, 45, 46, 47, 48, 49, 50, 51, 52, 53, 54, 55, 56, 57, 58, 59, 60, 61, 62, 63, 64, 65, 66, 67, 68, 69, 70, 71, 72, 73, 74, 75, 76, 77], and four prospective studies [78, 79, 80, 81]. Six case‐control studies [82, 83, 84, 85, 86, 87], and seven cross‐sectional studies [88, 89, 90, 91, 92, 93, 94] were included. Of the included studies, 28 were conducted in hospital settings [22, 23, 26, 32, 34, 36, 38, 43, 44, 45, 47, 52, 53, 60, 62, 67, 69, 70, 73, 76, 78, 79, 82, 85, 87, 88, 91, 92], and 51 were population‐based [4, 6, 18, 19, 20, 21, 24, 25, 26, 28, 29, 30, 31, 33, 35, 37, 39, 40, 41, 46, 48, 49, 50, 51, 52, 53, 54, 55, 56, 57, 58, 59, 61, 63, 64, 65, 66, 67, 68, 71, 72, 74, 75, 77, 80, 81, 84, 89, 90, 93, 94]. Eleven studies were conducted in Australia/New Zealand [25, 31, 33, 39, 41, 44, 55, 56, 75, 76, 94], 38 in North America [6, 21, 22, 23, 24, 26, 27, 29, 30, 32, 34, 35, 37, 40, 42, 43, 46, 47, 48, 49, 50, 51, 53, 54, 57, 58, 66, 67, 68, 69, 70, 71, 72, 80, 90, 91, 93], 27 in Europe [4, 18, 19, 20, 36, 38, 45, 59, 60, 61, 62, 63, 64, 65, 73, 77, 78, 79, 81, 82, 83, 84, 85, 87, 88, 89, 92], two in Israel [74, 86] and one in South Korea [28]. The 79 included studies incorporated data spanning a broad timeframe, from 1979 to 2021, and report 169 579 388 women, with 3 012 436 reported as having a PPH. Varying definitions of PPH were used: 16 studies defined PPH as > 500 mL blood loss [19, 20, 25, 35, 39, 45, 55, 59, 60, 61, 75, 76, 78, 92], 11 studies used > 1000 mL blood loss [33, 36, 43, 44, 46, 50, 53, 63, 64, 65, 70], seven studies used > 1500 mL blood loss [4, 18, 31, 47, 79, 85, 87] and one study used > 2000 mL blood loss [37]. Other definitions of PPH included > 500 mL after vaginal birth and > 750 mL at caesarean birth [41], > 500 mL after vaginal birth and > 1000 mL at caesarean birth [22, 26, 52, 74, 80] and a postnatal haemoglobin drop of 4 g/dL [90]. One study based on its case definition of intervention (i.e., use of uterotonics, blood transfusion or surgical management of PPH) [34], 12 studies used International Classification of Diseases and Related Health Problems 9th revision or 10th revision (ICD‐9 or ICD‐10) codes [6, 21, 24, 28, 30, 40, 42, 47, 57, 66, 71, 91], one study defined cases as those requiring transfer from midwifery led to obstetric care for PPH [39] and 23 studies in which the definition of PPH was not explicitly described [23, 27, 29, 32, 37, 49, 51, 54, 56, 58, 62, 67, 68, 69, 72, 75, 76, 77, 81, 83, 84, 86, 88, 93].

### 3.2 Quality Assessment

A detailed study quality assessment is presented in Tables S2–S4. The methodological quality of the included studies varied, with 25 studies assessed as low/poor quality [26, 27, 29, 32, 38, 45, 46, 50, 56, 57, 60, 66, 68, 71, 73, 75, 82, 83, 85, 86, 87, 88, 90, 92, 94], four studies of fair quality [25, 43, 89, 91] and 50 studies assessed as good quality [4, 6, 18, 19, 20, 21, 22, 23, 24, 28, 30, 31, 33, 34, 35, 36, 39, 40, 41, 42, 44, 47, 48, 49, 51, 53, 54, 55, 58, 59, 61, 62, 63, 64, 65, 67, 68, 69, 70, 72, 74, 76, 77, 78, 79, 80, 81, 84]. Funnel plots were used to assess for publication bias (Figures S1–S4). There was evidence of publication bias across all analyses.

### 3.3 Pooled Analysis of Ethnicity and Postpartum Haemorrhage Outcomes

When compared with White women or women from the majority ethnic group within the study, women from an ethnic minority background had an increased risk of PPH. This effect was seen across Black women (OR 1.16, 95% CI 1.09, 1.23), Asian women (OR 1.33, 95% CI 1.27, 1.39), and Hispanic women (OR 1.20, 95% CI 1.12, 1.29). For some studies, due to the way in which maternal ethnicity was described, a pooled effect estimate was calculated for minority ethnic groups compared to the majority ethnic group (White, European, or native‐born). This also demonstrated an increased risk of PPH among minority ethnic groups (OR 1.13, 95% CI 1.03, 1.24).

### 3.4 Subgroup Analysis by Study Continent

**FIGURE 2:**
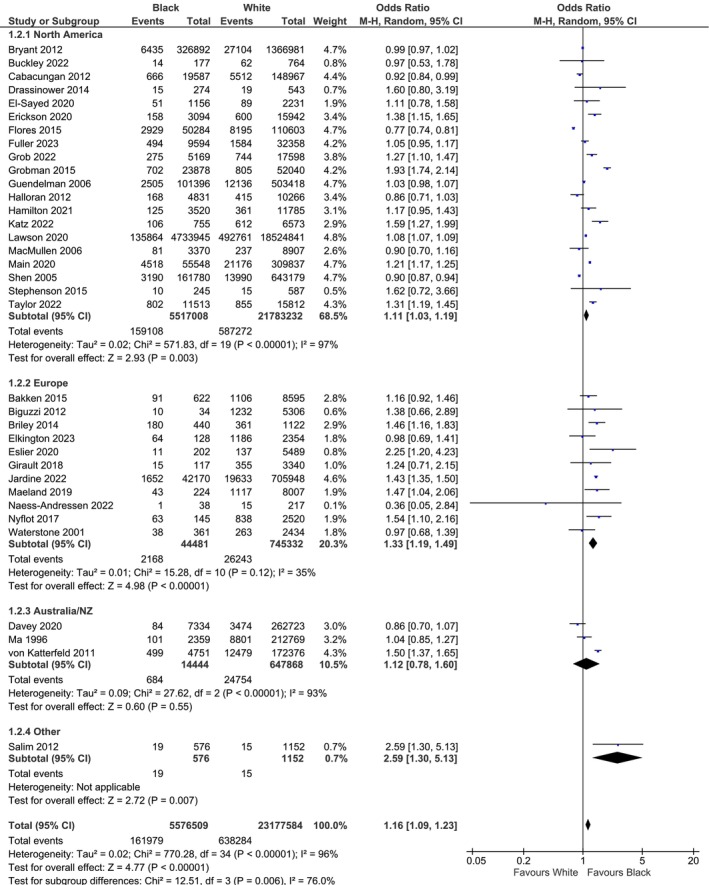
Risk of postpartum haemorrhage in White versus Black ethnicity, stratified by study continent. CI indicates confidence interval.

Subgroup analysis was performed to consider the effect of study continent (Table S5), where the difference in practice between settings may be expected, and study populations may be more homogeneous. Black women consistently show a higher risk of PPH across all geographic regions (Figure 2). Notably, European studies (OR 1.33, 95% CI 1.19, 1.49) displayed less heterogeneity than other regions (*I* ^2^ = 35%). For Asian women, the increased risk of PPH was most marked in studies conducted in Australia/New Zealand (Figure S5). Hispanic women exhibit an elevated risk of PPH in North American and European studies (Figure S6). For minority group women, the disparity was most marked in Australian/New Zealand studies, where the minority group usually represented indigenous women versus those of European origin (Figure S7).

High statistical heterogeneity is noted across the subgroup analyses performed, particularly for studies from North America and Australia/New Zealand. Leave‐one‐out analysis did not reveal any substantial differences in the *I* ^2^ statistic.

### 3.5 Subgroup Analysis for Time Period

To explore temporal trends, the effect over defined time periods (years 1990–1999, 2000–2009 and 2010–2019) was analysed (Table 1). The risk of PPH appeared to increase over time for Black, Hispanic and minority ethnic group women. To further explore this, stratified analysis by study continent and time period was conducted (Table S6).

**TABLE 1:** Subgroup analysis with regard to time period. Values are represented as odds ratio (95% confidence interval). *N* = number of studies. *I* ^2^ statistic as a measure of heterogeneity.

	1990–1999	2000–2009	2010–2019
White vs. Black	1.05 (0.90, 1.23) *N* = 6 *I* ^2^ = 95%	1.11 (1.01, 1.21) *N* = 18 *I* ^2^ = 95%	1.30 (1.17, 1.45) *N* = 13 *I* ^2^ = 89%
White vs. Asian	1.47 (1.21, 1.79) *N* = 7 *I* ^2^ = 98%	1.24 (1.19, 1.30) *N* = 15 *I* ^2^ = 94%	1.39 (1.16, 1.66) *N* = 9 *I* ^2^ = 97%
White vs. Hispanic	0.95 (0.85, 1.07) *N* = 3 *I* ^2^ = 94%	1.30 (1.17, 1.44) *N* = 12 *I* ^2^ = 98%	1.24 (1.08, 1.43) *N* = 5 *I* ^2^ = 76%
Majority vs. Minority	1.13 (0.88, 1.46) *N* = 5 *I* ^2^ = 98%	0.95 (0.87, 1.04) *N* = 5 *I* ^2^ = 96%	2.29 (1.34, 3.91) *N* = 5 *I* ^2^ = 94%

In North America, the risk of PPH increased for Black, Asian and Hispanic women over time. This is also seen in Europe for Black and minority ethnic group women. Conversely, in Europe, the risk for Asian women appears to decrease over time, although this was demonstrated in a small number of studies.

### 3.6 Subgroup Analysis for Potential Confounding Factors

Subgroup analyses for confounding factors were performed to consider the effect of maternal, foetal and birth factors that may influence the risk of PPH.

Table 2 demonstrates that after adjusting for clinical factors, Black, Asian, Hispanic and minority ethnic group women show a persistent increased risk of PPH. Specifically, Black and Asian women have consistently higher odds of PPH compared to White women, statistically significant for age, BMI and foetal macrosomia in particular. High heterogeneity and limited studies for some comparisons warrant cautious interpretation, suggesting other factors and methodological variations may contribute to these differences.

**TABLE 2:** Sensitivity analysis about risk factors for PPH. Values are represented as odds ratios (95% confidence interval). *N* = number of studies. *I* ^*2*^ statistic as a measure of heterogeneity.

	White vs. Black	White vs. Asian	White vs. Hispanic	Majority vs. Minority
Age	1.18 (1.04, 1.33) *N* = 12 *I* ^2^ = 96%	1.36 (1.21, 1.52) *N* = 13 *I* ^2^ = 95%	1.25 (1.05, 1.47) *N* = 7 *I* ^2^ = 98%	1.09 (0.97, 1.23) *N* = 6 *I* ^2^ = 97%
BMI	1.30 (1.13, 1.50) *N* = 6 *I* ^2^ = 72%	1.38 (1.10, 1.73) *N* = 6 *I* ^2^ = 97%	0.93 (0.21, 4.15) *N* = 2 *I* ^2^ = 89%	1.16 (1.01, 1.33) *N* = 4 *I* ^2^ = 79%
Parity	1.26 (1.00, 1.59) *N* = 5 *I* ^2^ = 98%	1.38 (1.17, 1.62) *N* = 8 *I* ^2^ = 96%	1.44 (0.71, 2.91) *N* = 2 *I* ^2^ = 99%	1.14 (0.99, 1.31) *N* = 5 *I* ^2^ = 98%
Medical comorbidities, e.g., hypertension, diabetes	1.11 (0.92, 1.35) *N* = 8 *I* ^2^ = 98%	1.36 (1.17, 1.57) *N* = 9 *I* ^2^ = 96%	1.23 (0.99, 1.53) *N* = 5 *I* ^2^ = 98%	1.07 (0.96, 1.20) *N* = 5 *I* ^2^ = 97%
Induction/labour augmentation	1.19 (0.83, 1.71) *N* = 6 *I* ^2^ = 97%	1.51 (0.31, 1.74) *N* = 7 *I* ^2^ = 94%	0.77 (0.28, 2.12) *N* = 2 *I* ^2^ = 77%	1.65 (1.32, 2.07) *N* = 1 *I* ^2^ = *N*/A
Mode of birth	1.06 (0.88, 1.27) *N* = 9 *I* ^2^ = 97%	1.41 (1.28, 1.54) *N* = 10 *I* ^2^ = 90%	1.08 (0.87, 1.34) *N* = 4 *I* ^2^ = 98%	1.38 (1.02, 1.87) *N* = 2 *I* ^2^ = 85%
Foetal macrosomia	1.47 (1.14, 1.88) *N* = 4 *I* ^2^ = 91%	1.50 (1.25, 1.80) *N* = 5 *I* ^2^ = 88%	2.07 (1.82, 2.36) *N* = 1 *I* ^2^ = *N*/A	1.21 (1.14, 1.28) *N* = 1 *I* ^2^ = *N*/A

### 3.7 Pooled Effect Estimates for Ethnicity and Severe Maternal Morbidity From Haemorrhage

A pooled analysis to estimate the combined effect of haemorrhage‐related SMM was conducted (Table S7). This integrated data on ITU admission, blood transfusion, peripartum hysterectomy, and SMM. Black (OR 1.57 95% CI (1.25, 1.98)), Asian (OR1.39 95% CI (1.25, 1.54)) and Hispanic (OR1.30 95% CI (1.18, 1.42)) women all showed a trend towards increased risk of SMM from haemorrhage, compared to White women.

### 3.8 Pooled Effect Estimate for Ethnicity and Maternal Mortality From Haemorrhage

Two studies [29, 57] provided data suitable for meta‐analysis (Figure S8). Women from ethnic minority groups showed a trend towards increased risk of maternal death from haemorrhage (OR 1.10 95% CI 0.73, 1.64) compared to White women.

### 3.9 Narrative Summary for Studies Not Amenable to Meta‐Analysis

Eight studies [6, 42, 63, 78, 82, 84, 89, 93] reported the risk of PPH in different socioeconomic groups across North America, Europe, and Australia. Data were not amenable to meta‐analysis owing to the differences in the measurement of socioeconomic status. Four studies [63, 79, 82, 89] used deprivation indices, two studies [6, 42] categorised participants based on their income quartiles and another utilised social class by occupation [84]. One study [93] compared adverse pregnancy outcomes in women in stable or unstable housing, defined by ICD‐9 and ICD‐10 codes. Seven studies reported an increased risk of PPH for those living in the most deprived areas, within the lowest income or socioeconomic groups, or exposed to unstable housing. Prick et al. [63] reported no significant difference between women from the lowest and highest socioeconomic groups. Staniczenko et al. [93] found that women exposed to unstable housing were more likely to experience PPH than those in stable housing (RR 1.74, 95% CI 1.65, 1.83), and that Non‐Hispanic Black women were more likely to be exposed to unstable housing than Non‐Hispanic White women (29.8% and 11.5%, respectively). Fotovati et al. [42] studied women who had undergone in vitro fertilisation (IVF) and reported reduced odds of PPH for those with a higher socioeconomic status (SES).

Five studies that reported the incidence or risk of PPH could not be included in the meta‐analysis due to data limitations. While some studies, such as Davis et al. [33], found an increased risk of PPH among Asian and Pacific Islander women, others, like Kanthasamy et al. [52], did not find significant differences between ethnic groups. Additionally, a US study found that Black women experienced a larger increase in PPH rates following the introduction of a blended payment policy [68].

Three studies [32, 51, 77] reported a higher risk of SMM due to haemorrhage among minority ethnic women. This increased risk was observed in various settings. For instance, Black women in the US experienced higher rates of SMM both before and after quality improvement interventions [32].

Two studies reported higher haemorrhage‐specific maternal mortality rates in Black compared to White women [27, 71].

## 4 Discussion

### 4.1 Main Findings

This systematic review and meta‐analysis included 79 studies of over 165 million women, with more than 3 million women experiencing a PPH. We have consistently demonstrated an elevated risk of PPH, SMM from haemorrhage and maternal death from haemorrhage among ethnic minority women and those experiencing socioeconomic deprivation in HICs. We found that, compared to White women, Black, Asian, and Hispanic women had 16%, 33%, and 20% increased odds of PPH, respectively. The subgroup analyses offer further insight into the robustness of these findings in the face of potential confounding. Adjusted analyses for key risk factors suggest that the association between ethnicity and PPH is not fully explained by these confounders. This study reveals a concerning trend of increasing PPH risk over time, disproportionately impacting Black, Hispanic, and other minority ethnic women, highlighting persistent health inequalities. The cause—widening disparity or a broader increase exacerbating existing vulnerabilities—remains unclear.

### 4.2 Strengths and Limitations

This is the largest systematic review examining the association between ethnicity, SES, and PPH risk. By including a larger number of studies and considering socioeconomic factors, this review provides a more comprehensive understanding of the issue.

There are several limitations to this review, notably high heterogeneity among studies included for meta‐analysis. This may be due to differences in study design, research methodology, study populations, and others. To explore this, leave‐one‐out analysis was performed, but did not yield any substantial differences in the *I* ^2^ statistic. Subgroup analyses revealed higher heterogeneity in studies from North America and Australia/New Zealand compared to Europe. Inclusion of studies focusing on specific high‐risk populations (such as multiple pregnancy, obesity, multiparity and medical co‐morbidities) may have introduced bias.

The lack of a standardised PPH definition may have introduced bias and limited the reliability of the findings, with 11 different definitions used across the included studies. Acknowledging heterogeneity in PPH definitions, the decision to pool data was made to synthesise the overall evidence regarding the association with ethnicity and socioeconomic status. Analysing these separately may have precluded meta‐analysis. PPH is defined as blood loss of 500 mL or more from the genital tract after birth by the World Health Organisation (WHO) [95], whereas the PPH core outcome set [15] recommends blood loss > 1000 mL as a key outcome measure. In future, the adoption and consistent application of the core outcome set for PPH should be considered, and researchers must clearly define PPH in their work. Similarly, there is a lack of a standardised definition for SMM, which presents a significant challenge in the comparability of findings. International organisations, such as the WHO [96] and Centers for Disease Control and Prevention (CDC) [97], have proposed standardised criteria to address this.

The limited number of studies reporting PPH per socioeconomic group hinders a comprehensive understanding of its impact. Measuring socioeconomic deprivation is complex and context specific, and varying measures across different countries further complicate the analysis and limit the ability to draw robust conclusions.

The diverse classification systems used to describe ethnicity and race across studies, such as ‘Black’, ‘White’, ‘Asian’, ‘Hispanic,’ or maternal country of origin, hindered the comparability of findings and posed challenges for meta‐analysis. In some countries, such as France and Germany, the collection of ethnicity data is restricted [98], posing significant challenges for researchers seeking to understand health disparities. For studies reporting maternal country of birth, we made assumptions about maternal ethnicity. For example, women from Sub‐Saharan Africa were categorised as Black, and those from Europe as White, to enable meta‐analysis. The use of broad categories such as ‘Black’ or White’ can mask important variations within these groups, leading to potential bias in the results. Future research should focus on collecting more granular ethnicity data and consider including measures such as migration status, language barriers, and acculturation. This would enable the exploration of social and cultural factors that may contribute to differential PPH risk beyond ethnicity and SES.

### 4.3 Interpretation

Ultimately, race, ethnicity, and socioeconomic deprivation are social constructs that are contextually generated [99]. While they are difficult to measure consistently across international studies, they are crucial for understanding health disparities [100]. There is evidence that women from ethnic minority groups are more likely to be exposed to socioeconomic deprivation [3, 79]. Black and Hispanic women are more likely to live in areas of concentrated poverty, which are likely to have significant health impacts [101]. A deeper understanding of these disparities requires further research that considers the intersectionality of ethnicity and SES. This intersectionality could not be assessed in this study.

The causes of PPH disparities are complex, and future research should investigate the interplay of various factors, including individual biological predispositions. For example, ethnic minorities higher prevalence of fibroids, pre‐eclampsia, anaemia, and gestational diabetes may contribute to their increased PPH risk [102, 103, 104]. However, this exploration must be balanced with an examination of systemic racism and unequal access to care, avoiding harmful ‘broken bodies’ narratives [105].

Ethnic minority women may face other barriers, such as limited access to healthcare, and language difficulties may impact the opportunity to access timely and adequate care. Racism in healthcare contributes to poorer maternal outcomes [105]. Stigma and discrimination can hinder minority ethnic groups' access to equitable maternity care, potentially increasing adverse outcomes like PPH due to factors such as reduced trust and differential treatment [106]. Reflecting broader societal issues, the stigma and bias observed within healthcare settings likely mirror the higher rates of racism and discrimination faced by minority groups in the wider community [107]. Persistent exposure to racism, stigma, and socioeconomic hardship can increase allostatic load—the body's wear and tear from chronic stress—and physiological weathering in marginalised groups, potentially raising their PPH risk due to cumulative bodily strain [108].

Implicit biases within the healthcare system can affect the quality of care provided to women. A lack of insight among healthcare professionals may inhibit communication, shared decision making, and overall patient care. The requirement for personalised, respectful, and sensitive maternity care for all women has been repeatedly highlighted in reports assessing maternity care in the UK [109, 110]. MBRRACE noted that Black women were overrepresented among the group of women who failed to receive individualised and culturally sensitive care [7].

## 5 Conclusion

This systematic review and meta‐analysis of 79 studies examined over 165 million women, more than 3 million of whom experienced a PPH. We found that there is an increased risk of PPH for Black, Asian, Hispanic, and women from minority ethnic groups compared to White women, and for women exposed to socioeconomic deprivation. There was high heterogeneity among included studies, which may impact the results. The identification of an increased risk of PPH among ethnic minority and deprived women is a critical first step towards addressing this issue. A comprehensive understanding requires a balanced exploration of biological, social, and systemic factors, taking care to develop interventions that target these underlying causes rather than focusing on individual risk, avoiding potential racial bias. Healthcare systems should actively tackle racism to foster equitable care and lessen the impact of allostatic load on marginalised individuals through high‐quality, culturally competent services. Furthermore, involving women and community members in research is vital for developing effective interventions and improving maternity outcomes. Strategies that target improved data collection on maternal outcomes disaggregated by ethnicity and SES are necessary. Ethnic minorities and women from deprived backgrounds are frequently underrepresented in research and therefore in policymaking, and adequate inclusion in research has become a priority for research funders such as the National Institute for Health and Care Research (NIHR) in the UK and the CDC [111, 112].

## Author Contributions

A.E. conceptualised and designed the study, performed screening, data collection and analysis, and wrote the manuscript. J.C. and R.I. performed literature searches, reviewed and edited the manuscript. G.A., M.J., E.M., and R.I. performed screening, reviewed, and edited the manuscript. M.P.R. and N.C. curated data, verified the dataset, performed data analysis, and reviewed and edited the manuscript. B.A.‐W. and S.B. provided methodological advice, and reviewed and edited the manuscript. B.K., W.P.‐S., and P.W. conceptualised and designed the study, reviewed and edited the manuscript, and provided supervision to A.E.

## Ethics Statement

The authors have nothing to report.

## Conflicts of Interest

The authors declare no conflicts of interest.

## Supporting information


Appendix S1.

## Data Availability

The data that support the findings of this study are available from the corresponding author upon reasonable request.
