# Supplementary material for: Effects of Race, Ethnicity and Socioeconomic Deprivation on Postpartum Haemorrhage in High‐Income Countries: A Systematic Review and Meta‐Analysis
Source: BJOG. 2025 Jul 9;132(13):1983–95. doi: 10.1111/1471-0528.18278 (PMC12592775; doi:10.1111/1471-0528.18278)
Supplement: Supplementary file 1 — Appendix S1. [file BJO-132-1983-s001.docx]

**Supplementary Material**

**Appendix S1: Search terms and search strategy for A. MEDLINE, B. Embase, C. CINAHL, D. Google Scholar**

1. Ovid MEDLINE

1 Postpartum Hemorrhage/

2 ((postpartum or postnatal or childbirth or parturition or birth or labour or labor or delivery or post-delivery) adj2 (bleed* or haemorrhage or hemorrhage or (blood adj2 loss))).ti,ab.

3 PPH.ti,ab.

4 1 or 2 or 3

5 exp population groups/

6 (ethnicity or "people of colo?r" or bipoc or bame or bme).ti,ab.

7 ((ethnic* or race or racial or national*) adj2 (minorit* or group* or origin or identity or cohort* or population*)).ti,ab.

8 (migrant* or immigrant* or asylum or refugee*).ti,ab.

9 5 or 6 or 7 or 8

10 (deprivation or poverty or disadvantage*).ti,ab.

11 ("index of multiple deprivation" or imd or ses).ti,ab.

12 ((social or socioeconomic or economic) adj2 (hierarchy or mobility or status or group or factors)).ti,ab.

13 exp Socioeconomic Factors/

14 10 or 11 or 12 or 13

15 9 or 14

16 4 and 15

1. EMBASE

1 exp postpartum hemorrhage/

2 ((postpartum or postnatal or childbirth or parturition or birth or labour or labor or delivery or post-delivery) adj2 (bleed* or haemorrhage or hemorrhage or (blood adj2 loss))).ti,ab.

3 PPH.ti,ab.

4 1 or 2 or 3

5 exp ethnic group/ or exp minority group/

6 (ethnicity or "people of colo?r" or bipoc or bame or bme).ti,ab.

7 ((ethnic* or race or racial or national*) adj2 (minorit* or group* or origin or identity or cohort* or population*)).ti,ab.

8 (migrant* or immigrant* or asylum or refugee*).ti,ab.

9 5 or 6 or 7 or 8

10 (deprivation or poverty or disadvantaged).ti,ab.

11 ("index of multiple deprivation" or imd or ses).ti,ab.

12 ((social or socioeconomic or economic) adj2 (hierarchy or mobility or status or group or factors)).ti,ab.

13 exp household economic status/ or exp poverty/ or exp socioeconomic vulnerability/

14 10 or 11 or 12 or 13

15 9 or 14

16 4 and 15

1. CINAHL
2. (MH “Postpartum Hemorrhage”)
3. ((postpartum or postnatal or childbirth or parturition or birth or labour or labor or delivery or post-delivery) N2 (bleed* or haemorrhage or hemorrhage or (blood N2 loss)))
4. PPH
5. S1 OR S2 OR S3
6. (MH “Population +”)
7. (ethnicity or “people of colo?r” or bipoc or bame or bme)
8. ((ethnic* or race or racial or national*) N2 (minorit* or group* or origin or identity or cohort* or population*))
9. (migrant* or immigrant* or asylum or refugee*)
10. S5 OR S6 OR S7 OR S8
11. (deprivation or poverty or disadvantage*)
12. (“index of multiple deprivation” or imd or ses)
13. ((social or socioeconomic or economic) N2 (hierarchy or mobility or status or group or factors))
14. (MH “Socioeconomic Factors+”)
15. S10 OR S11 OR S12 OR S13
16. S9 OR S14
17. S4 AND S15
18. Google scholar search:

allintext:("Postpartum Hemorrhage" OR "postpartum haemorrhage") around (2) (minority OR minorities OR poverty OR ethnic OR racial OR "economic status" OR "social mobility" OR "social deprivation" OR "social class") -medline -embase -cochrane

**Appendix S2: Newcastle Ottawa Scale Risk of Bias Assessment Tool**

Newcastle Ottawa Scale for Cohort Studies

SELECTION

1. Representativeness of the exposed cohort
   1. Truly representative of the average woman giving birth in the population*
   2. Somewhat representative of the average woman giving birth in the population*
   3. Selected group of patients
   4. No description of the derivation of the cohort
2. Selection of the non-exposed cohort
   1. Drawn from the same population as the exposed cohort*
   2. Drawn from a different population
   3. No description of the non-exposed cohort
3. Ascertainment of exposure
   1. Secure records (e.g. medical records)*
   2. Structured interview*
   3. Written self report
   4. No description
4. Demonstration that outcome of interest was not present at the start of the study
   1. Yes*
   2. No

COMPARABILITY

1. Comparability of cohorts on the basis of design or analysis
   1. Study controls for maternal/feta/birth characteristics*
   2. Study controls for any additional factor*
   3. None

OUTCOME

1. Assessment of outcome
   1. Independent blind assessment*
   2. Record linkage*
   3. Self report
   4. No description
2. Was follow up long enough for outcomes to occur
   1. Yes*
   2. No
3. Adequacy of follow up of cohorts
   1. Complete follow up – all subject accounted for*
   2. Subjects lost to follow up unlikely to produce bias, or description provided of those lost*
   3. Follow up rate < 80% and no description of those lost
   4. No statement

Newcastle Ottawa Scale for Case-control Studies

SELECTION

1. Is the case definition adequate?
   1. Yes, with independent validation*
   2. Yes, eg record linkage or based on self-reports
   3. No description
2. Representativeness of the cases
   1. Consecutive or obviously representative series of cases*
   2. Potential for selection bias or not stated
3. Selection of controls
   1. Community/population controls*
   2. Hospital controls
   3. No description
4. Definition of controls
   1. No history of disease*
   2. No description of source

COMPARABILITY

1. Comparability of cases and controls on the basis of the design or analysis
   1. Study controls for maternal/fetal/birth characteristics*
   2. Study controls for any additional factor*

EXPOSURE

1. Ascertainment of exposure
   1. Secure record (e.g. medical record)*
   2. Structure interview interview where blind to case/control status*
   3. interview not blinded to case/control status
   4. written self report or medical record only
   5. no description
2. Same method method of ascertainment for cases and controls
   1. Yes*
   2. No
3. Non-Response rate
   1. same rate for both groups*
   2. non respondents described
   3. rate different and no designation

Adapted Newcastle-Ottawa Scale for Cross-Sectional Studies

SELECTION

1. Representativeness of the sample
   1. sample was truly representative of the average in the target population (e.g. all subjects or random sampling)*
   2. Somewhat representative (non-random sampling)*
2. Sample size
   1. Sample size justified and satisfactory*
3. Non-included subjects
   1. Comparability between included and non-included subjects established, and if inclusion rate is satisfactory*

COMPARABILITY

1. Subjects in different groups are comparable based on study design or analysis. Confounding factors are controlled
   1. Study controls for fetal/maternal/birth characteristics*
   2. Study controls for any additional factor*

OUTCOME

1. Assessment of outcome
   1. Independent blind assessment*
   2. Record linkage*
   3. Self report
   4. No description
2. Statistical test
   1. Statistical test clearly described and appropriate, measurement of association presented including confidence intervals and probability level (P value)*

**Table S1: Characteristics of Included Studies**

| **Study Author** | **Year** | **Country** | **Study Design** | **Purpose/Objectives** | **Sample Size** | **Population Characteristics** | **Primary Outcome** | **Data Collection Time Frame** |
| --- | --- | --- | --- | --- | --- | --- | --- | --- |
| **Almeida et al.^16^** | 2014 | Portugal | Cross-sectional study | To evaluate differences in obstetric care between immigrant and native women in a country with free access to healthcare | 277 | Portuguese – 67·87%  Migrants – 32·13% | Timing of first prenatal visit, mode of birth, pregnancy complications including perineal laceration and postpartum haemorrhage | 2012 |
| **Al-Zirqi et al.^17^** | 2008 | Norway | Retrospective cohort study | To determine the prevalence, causes, risk factors and  acute maternal complications of severe obstetric haemorrhage | 307415 | European – 89·52%  Middle Eastern – 2·11%  South-East Asian – 1·99%  Other – 6·36% | Severe obstetric haemorrhage (>1500ml blood loss or blood transfusion) | 1999-2004 |
| **Bakken et al.^18^** | 2015 | Norway | Retrospective cohort study | To compare the obstetric  outcomes of immigrants and ethnic Norwegians in a low risk setting | 11540 | Norway – 59·15%  W·Europe/USA/Aus/NZ – 15·33%  E.Europe – 7·66%  Latin America & Caribbean – 1·58%  Asia – 10·89% Africa – 5·39% | Obstetric outcomes including onset of labour, mode of birth, episiotomy, postpartum haemorrhage, epidural analgesia, labour dystocia, preterm birth, neonatal outcomes. | 2006-2010 |
| **Bakken et al.^19^** | 2017 | Norway | Retrospective cohort study | To examine if there are differences in the  background characteristics and obstetric outcomes between  first- and second-generation Pakistani immigrants in a low risk  birth setting | 8524 | Norweigan – 96·63%  Pakistani – 3·37% | Labour onset, epidural analgesia, labour dystocia,  episiotomy, vaginal/operative delivery, postpartum  haemorrhage, preterm birth, birth weight, transfer to a  neonatal intensive care unit, and neonatal jaundice. | 2006-2013 |
| **Biguzzi et al.^20^** | 2012 | Italy | Prospective cohort study | To define the prevalence of postpartum haemorrhage and associated risk factors after vaginal birth  and to develop a risk model that improves postpartum haemorrhage prediction. | 6011 | Caucasian – 88·27%  Hispanic – 7·47%  Asian – 3·69%  African – 0·57% | Postpartum haemorrhage (> 500mls blood loss) | 2007-2009 |
| **Briley et al.^21^** | 2014 | UK | Prospective cohort study | To quantify reporting errors, measure incidence of  postpartum haemorrhage (PPH) and define risk factors for PPH  (≥500 ml) and progression to severe PPH (≥1500 ml). | 1895 | White -59·2%  Black – 23·2%  Asian – 10·3%  Mixed – 2·6%  Unknown – 4·7% | Incidence and risk factors for PPH and  progression to severe PPH. | 2008-2009 |
| **Brown et al.^22^** | 2021 | UK | Cross-sectional study | To assess the  relationship between deciles of area-level  deprivation and  seven adverse pregnancy outcomes | 64699 | Index of multiple deprivation deciles:  Decile 1 most deprived used as reference category·  Decile 10 least deprived·  (Percentage of population in each decile not given in paper) | Small  for gestational age, large for gestational age,  preterm birth; third-degree  or fourth-degree  perineal  tear, major postpartum haemorrhage, a  lower Apgar score at 5 min and emergency caesarean  section. | 2014-2019 |
| **Bryant et al.^23^** | 2012 | USA | Retrospective cohort study | To examine racial/ethnic variation in rates of atonic PPH | 2,488,974 | White – 54·92%  Black – 13·13%  Hispanic -26·63%  Asian/Pacific Islander – 5·30% | PPH from uterine atony, PPH from uterine  atony requiring transfusion of packed red blood cells, and PPH from uterine atony requiring hysterectomy | 2005-2008 |
| **Buckley et al.^24^** | 2022 | USA | Retrospective cohort study | To examine the ability of a prediction model to predict the chance of successful VBAC, secondary goal to determine whether race/ethnicity was associated with increased risk of morbidity | 1241 | Non-Hispanic White – 61·6%  Hispanic – 18·3%  Non-Hispanic Black – 14·3%  Asian/Other – 5·9% | Pregnancy, labour and birth complications | 2016-2019 |
| **Butwick et al.^25^** | 2014 | USA | Retrospective cohort study | To investigate  patient characteristic, obstetric, anaesthetic, and intrapartum  risk factors for severe haemorrhage-related morbidity  among women who experience UA during Caesarean delivery | 2294 | Africa-American – 22·01%  Caucasian – 36·75%  Hispanic – 35·70%  Other – 5·57% | Haemorrhage-related morbidity e.g. blood transfusion, caesarean hysterectomy, uterine/hypogastric artery ligation, ITU admission | 1999-2002 |
| **Cabacungan et al.^26^** | 2012 | USA | Retrospective cohort study | To examine racial/ethnic disparities in maternal morbidity during labour and  delivery | 206428 | White – 72·16%  African- American – 9·49%  Hispanic – 8·20%  Native American - 1·07%  Others – 1·78% | (1) types of maternal  morbidities (MM) and (2) the number of MM (MM refers to obstetric complications during labour and delivery | 1998-2001 |
| **Calje et al.^27^** | 2023 | New Zealand | Retrospective cohort study | To report the incidence of postpartum anaemia (PPA) in three district health board regions  and describe current management of moderate to severe PPA, including  by ethnicity | 8849 | Maori – 20%  Pacific – 17%  Indian – 10%  Asian – 10%  Middle Eastern/Latin American/African – 3%  European – 40% | Incidence of postpartum anaemia and describe management of women with Hb < 90 | 2019 |
| **Chalouhi et al.^28^** | 2015 | USA | Retrospective cohort study | To assess whether Native American women have an increased risk of postpartum haemorrhage after vaginal delivery. | 1062 | Native American – 70·72%  Hispanic – 7·82%  White – 18·27%  Middle Eastern – 1·13%  Asian – 1·22%  Black – 0·85% | Incidence of postpartum haemorrhage (visual estimation > 500mls blood loss) | 2009-2012 |
| **Chichakli et al.^29^** | 1999 | USA | Retrospective observational study | To study trends and examine risk factors for  pregnancy-related mortality due to haemorrhage. | 763 | White  Black  (percentages of study population in each group not given in paper) | Pregnancy related mortality ratio due to haemorrhage | 1979-1992 |
| **Cho et al.^30^** | 2022 | South Korea | Retrospective cohort study | To evaluate the pregnancy outcomes  of immigrant women in Korea. | 4439778 | Korean women – 96·19%  Immigrant women – 3·81% | Adverse pregnancy outcomes including  gestational diabetes, preeclampsia, caesarean section, placental abnormalities,  and PPH | 2007-2016 |
| **Creanga et al.^31^** | 2012 | USA | Retrospective observational study | To compare trends in and causes of pregnancy-  related mortality by race, ethnicity, and nativity  from 1993 to 2006 | 7487 | White – 40·04%  Black – 39·97%  Hispanic – 15·91%  Asian/Pacific Islander – 4·07% | Pregnancy related mortality ratios for each race, ethnicity and nativity group | 1993-2006 |
| **Creanga et al.^32^** | 2014 | USA | Retrospective observational study | To examine racial and ethnic disparities in severe disparities in severe maternal morbidity during delivery hospitalisations in the United States | 3476392 | Non-Hispanic White – 42·72%  Non- Hispanic Black – 12·50%  Hispanic – 32·81%  Asian/Pacific Islander – 7·13%  American Indian/Alaskan Native – 0·59% | Rate of severe maternal morbidity during delivery hospitalisations by race/ethnicity | 2008-2010 |
| **Davey et al.^33^** | 2020 | Australia | Retrospective cohort study | To identify risk factors for severe PPH | 364706 | Australia – 67·8%  Americas – 1·3%  . Africa & Middle East – 3·7%  Sub-saharan Africa – 2·0%  Asia – 17·2%  Europe – 5·1%  Oceania & Antarctica – 2·8% | Incidence of severe PPH (defined as > 1500mls blood loss) | 2009-2013 |
| **Davidson et al.^34^** | 2022 | USA | Before and after study | To evaluate the impact of  quality improvement and patient safety initiatives  and data disaggregation on racial disparities in severe  maternal morbidity from haemorrhage | 13659 | Non-Hispanic White – 34·44%  Non-Hispanic Black – 19·98%  Hispanic – 37·6% Non-Hispanic Asian – 7·42% Other – 0·56% | Rate of severe maternal morbidity from haemorrhage by race and ethnicity | 2015-2020 |
| **Davis et al.^35^** | 2012 | New Zealand | Retrospective cohort study | To investigate the effect of place of  birth on the risk of postpartum haemorrhage and the  effect of mode of management of the third stage of  labour on severe postpartum haemorrhage | 16453 | NZ European (used as reference group)  Maori  Pacific Islander  Asian  Other  (percentages of study population in each group not given in paper) | Incidence of PPH >1000mls and mode of third stage management | 2006-2007 |
| **Drassinower et al.^36^** | 2014 | USA | Secondary analysis of prospective observational study | To determine the relationships  between maternal race and obstetric outcomes in twin  gestations by planned mode of delivery | 1009 | White – 54%  African American – 27%  Hispanic – 13% Asian – 3%  Other/unknown – 3% | Unplanned  caesarean section in the trial of labour group | 2002-2008 |
| **Elkington et al.^37^** | 2023 | UK | Case-control study | To estimate the incidence of, and investigate risk factors for, postpartum haemorrhage requiring transfer to obstetric care following birth in midwifery units in the UK and describe outcomes for women who experience PPH requiting transfer to obstetric care | 2976 | White – 79·10%  Asian – 10·99%  Black – 4·30%  Other – 5·61% | Estimated blood loss volume, cause and management of PPH, neonatal and maternal outcomes, OR (95%CI) to investigate risk factors for PPH requiring transfer to obstetric care | 2019-2020 |
| **El-Sayad et al.^38^** | 2020 | USA | Observational (secondary analysis of RCT – ARRIVE trial) | To evaluate characteristics associated with adverse outcomes in low-risk nulliparous women randomized to elective labour induction at 39 weeks of gestation or expectant management | 5007 | Non-Hispanic White – 44·56%  Non-Hispanic Black – 23·09%  Hispanic – 26·78%  Asian/Pacific Islander – 3·02%  Other – 2·56% | A composite of perinatal mortality and severe perinatal morbidity, 3rd or 4th degree perineal lacerations, and postpartum haemorrhage | 2014-2017 |
| **Erickson et al.^39^** | 2020 | USA | Retrospective cohort study | To identify  phenotypes of labour processes and  estimate the likelihood of PPH by phenotype, and analyse how maternal and fetal characteristics relate to PPH risk by phenotype | 24,729 | Non-Hispanic White – 64·5%  Non-Hispanic Black – 12·5%  Hispanic – 14·9%  Asian/Pacific Islander – 4·1% | Incidence of PPH (estimated blood loss > 500mlsafter vaginal birth and ICD-9 codes) | 2002-2008 |
| **Eslier et al.^40^** | 2020 | France | Retrospective cohort study | To compare changes in maternal and perinatal morbidity inequalities among migrant and  native women over time | 6036 | France – 89·17%  Europe – 1·77%  . Africa – 3·13%  Sub-Saharan Africa – 3·35%  Other – 2·63% | Severe maternal morbidity including severe pre-eclampsia, eclampsia, HELLP syndrome, placental abruption, uterine rupture, severe PPH (>1000mls blood loss) | 2008 and 2014 |
| **Estrada et al.^41^** | 2022 | USA | Retrospective cohort study | To describe racial/ethnic disparities  in severe maternal morbidity by using admission to an intensive care unit (ICU) as a marker | 1340 | Non-Hispanic White – 21·22%  Asian – 18·30%  Filipino – 19·67%  Hawaiian/Pacific Islander – 34·64% Hispanic – 3·25%  Non-Hispanic Black – 1·23%  Other – 1·69% | ITU admission by race/ethnicity and clinical characteristics and obstetric outcomes | 2012-2017 |
| **Fadel et al.^42^** | 2019 | UK | Retrospective cohort study | To review cases of a massive PPH and compare maternal characteristics  and differences in obstetric practice when blood loss > 3000mls | 184 | White – 33%  Asian – 10%  Black – 45%  Chinese/Other – 12% | Maternal characteristics, factors in obstetric practice,  estimated blood loss  and other maternal and neonatal outcomes | 2007-2014 |
| **Faulks et al.^43^** | 2023 | Australia | Retrospective cohort study | To examine the perinatal outcomes of women who experience social disadvantage | 1188872 | Index of relative disadvantage quintiles  1^st^ (most disadvantaged) – 15·5%  2^nd^ – 15·9%  3^rd^ – 20·3%  4^th^ – 22·4%  5^th^ (least disadvantaged, used as reference) – 25·9% | Incidence of maternal admission to intensive care unit, postpartum haemorrhage and caesarean section, perinatal mortality,  preterm birth, low birthweight, and admission to special care nursery/neonatal  intensive care unit. | 1999-2016 |
| **Flores et al.^44^** | 2015 | USA | Retrospective cohort study | To examine whether maternal asthma contributes to racial/ethnic differences in obstetric and neonatal complications | 208,899 | White – 52·95%  Black – 24·07%  Hispanic – 18·59% | Maternal demographics, medical history, obstetric and neonatal outcomes | 2002-2008 |
| **Ford et al.^45^** | 2007 | Australia | Retrospective cohort study | To determine whether changes in risk factors for postpartum haemorrhage over  time are associated with a rise in postpartum haemorrhage rates | 164133 | Australia – 73·47%  Overseas – 26·16%  Unknown – 0·36% | Incidence of PPH (defined as > 500ml following vaginal birth and > 750ml at caesarean birth) | 1994-2002 |
| **Fotovati et al.^46^** | 2024 | USA | Retrospective observational study | To evaluate if socioeconomic status has an impact on perinatal outcomes in in vitro fertilisation pregnancies | 10439 | Higher SES – 93·2%  Lower SES (defined as lowest 25% of individuals based on yearly family income) – 6·7% | Maternal outcomes of preterm prelabor rupture of membranes, preterm birth, placental abruption, caesarean delivery, operative vaginal delivery, spontaneous vaginal delivery, maternal infection, chorioamnionitis, hysterectomy, postpartum haemorrhage. Neonatal outcomes small for gestational age, intrauterine fetal death, congenital anomalies. | 2008-2014 |
| **Fuller et al.^47^** | 2023 | USA | Retrospective cohort study | To evaluate racial disparities in obstetrical outcomes in the Military Health System. | 68918 | White – 47%  Black – 13·9%  Asian/Pacific Islander – 4·5%  Native American/Alaskan Native – 0·7%  Other – 28·4%  Unknown – 5·8% | Incidence of caesarean section, postpartum haemorrhage and severe maternal morbidity by race and ethnicity from direct-care military hospitals | 2019-2021 |
| **Fyfe et al.^48^** | 2012 | New Zealand | Retrospective cohort study | To determine whether being overweight and obesity are independent risk factors for major postpartum haemorrhage | 11363 | European – 53·36%  Maori – 7·42%  Pacific Island – 8·95%  Asian – 27·36%  Other – 2·90% | Major primary postpartum haemorrhage  defined as blood loss > 1000mls within 24hrs of delivery | 2006-2009 |
| **Girault et al.^49^** | 2018 | France | Observational study (secondary analysis of TRACOR RCT) | To evaluate the incidence of undiagnosed abnormal postpartum blood loss  after vaginal delivery, identify the risk factors and compare them to those of postpartum  haemorrhage | 3917 | France – 81·6%  Europe – 3·6%  N. Africa – 6·3%  Sub-Saharan Africa – 3·0%  Asia – 1·8%  French overseas territories – 1·3%  Other – 1·9% | Occurrence of PPH or undiagnosed abnormal postpartum blood loss | 2010-2011 |
| **Grob et al.^50^** | 2022 | USA | Retrospective cohort study | To evaluate if universal access to healthcare, as seen in the military healthcare system, leads to similar rates of maternal morbidity regardless of racial or ethnic background | 36861 | White – 47·74%  Black – 14·02%  Asian/Pacific Islander – 4·82%  Other -28·16% | Incidence of PPH, severe maternal morbidity among women with PPH including and excluding blood transfusion | 2019-2020 |
| **Grobman et al.^51^** | 2015 | USA | Retrospective cohort study | To evaluate whether racial and ethnic disparities exist in obstetric care and adverse outcomes. | 109,208 | Non-Hispanic White – 48%  Non-Hispanic Black – 22%  Hispanic – 25%  Asian – 5% | Incidence of severe postpartum haemorrhage, peripartum infection, and severe perineal laceration at spontaneous vaginal delivery | 2008-2011 |
| **Guendelman et al.^52^** | 2006 | USA | Retrospective cohort study | To compare obstetric complications between White, Black, Asian and Latina women | 1426854 | Latinas – 51%  White – 35%  Black – 7%  Asian – 6·9% | Incidence of maternal morbidities (using ICD-9 codes) | 1996-1998 |
| **Guglielminotti et al.^53^** | 2022 | USA | Cross-sectional study | To assess the association between the use of labour neuraxial analgesia for vaginal  delivery and SMM. | 575 524 | Asian/Pacific Islander – 8·0%  Non-Hispanic Black – 15·4%  Hispanic – 18·2%  Non-Hispanic White – 44·9%  Other – 13·0% | Severe maternal morbidity, postpartum haemorrhage | 2020-2021 |
| **Guomundsdottir et al.^54^** | 2021 | Iceland | Prospective cohort study | To explore maternal and perinatal outcomes of migrant  women in Iceland | 92403 | Icelandic women – 91·17%  Migrant women – 8·83% | Onset of labour, augmentation, epidural, perineum support, episiotomy, mode of birth,  obstetric anal sphincter injury, postpartum haemorrhage, preterm birth, a 5-minute Apgar <7, neonatal intensive care unit admission and perinatal mortality | 1997-2018 |
| **Gyamfi-Bannerman et al.^6^** | 2018 | USA | Retrospective cohort study | To assess how race is  associated with adverse maternal outcomes in the setting of postpartum  haemorrhage. | 11260869 | Non-Hispanic White – 5·40%  Non-Hispanic Black – 13·67%  Hispanic – 21·23% Asian/Pacific Islander – 6·51%  Native American – 1·05%  Other – 4·63%  Unknown – 7·51% | Severe maternal morbidity as defined by the CDC | 2012-2014 |
| **Halloran et al.^55^** | 2012 | USA | Retrospective cohort study | To explore the trends in prepregnancy BMI for Black and White  teenagers over time and the association between elevated BMI and outcomes based on race | 15,097 | White 68%  Black 32% | Prevalence of elevated  prepregnancy BMI and the association between elevated prepregnancy  BMI and maternal and perinatal outcomes based on race | 1993-2006 |
| **Hamilton et al.^56^** | 2021 | USA | Retrospective cohort study | To investigate the hypothesis that universal access to healthcare leads to similar rates of maternal morbidity regardless of race/ethnicity | 15305 | White – 77%  Black – 23% | Severe maternal morbidity as defined by the CDC, postpartum haemorrhage > 1000mls blood loss | 2018-2019 |
| **Hartenbach et al.^57^** | 2020 | USA | Retrospective cohort study | To evaluate the peripartum transfusion rates for rural women compared to urban women in the United States | 3346816 | Non-Hispanic White – 56·9%  Non-Hispanic Black – 13·4%  Hispanic – 20·7%  Native American – 0·8%  Asian/pacific Islanders – 7·8% | Rate of blood transfusion | 2014-2016 |
| **Harvey et al.^58^** | 2017 | USA | Retrospective cohort study | To assess racial-ethnic differences in the prevalence of postpartum haemorrhage | 243,693 | Pacific Islander – 35·0%  Asian – 44·0%  White – 21·0% | Discharge diagnosis of PPH as defined by ICD-9 Codes | 1995-2013 |
| **Jardine et al.^4^** | 2022 | UK | Cohort study | To determine the association between ethnic group and risk of postpartum  haemorrhage in women giving birth | 981801 | White – 77·8%  South Asian – 11·8%  Black – 4·6%  Mixed – 1·8%  Other – 3·9% | Postpartum haemorrhage of ≥1500 ml | 2015-2017 |
| **Kanthasamy et al.^59^** | 2013 | UK | Retrospective case-control study | To compare the  demographic and obstetric outcomes between ethnic minority women and Caucasian British women | 296 | British born Caucasian – 50%  Turkish – 50% | Maternal and fetal demographics and outcomes | 2005-2006 |
| **Katz et al.^60^** | 2022 | USA | Retrospective cohort study | To compare postpartum blood loss and PPH in African American  and Hispanic parturients compared to other groups | 6040 | African American – 9·9%  Hispanic – 13·6%  Non African American/Hispanic – 76·5% | Median blood loss volumes in racial and ethnic groups in QBL and EBL cohorts | 2016-2017 |
| **Lawson et al.^61^** | 2020 | USA | Retrospective cohort study | To compare perioperative outcomes by patient race/ethnicity. | 35043246 | White – 52·9%  Black - 13·5%  Hispanic - 23·0%  Asian – 5·2%  Other – 5·4% | Peripartum hysterectomy,  in‐hospital mortality, perioperative complications, length of stay, and cost of hysterectomy | 2004-2014 |
| **Lucas et al.^62^** | 2021 | Australia | Cohort study | To assess associations of hyperglycemia in pregnancy with the risk of postpartum  Haemorrhage in a prospective cohort of Indigenous and non-Indigenous  women, compared with normoglycemia. | 1102 | Indigenous women – 47·64%  Non-indigenous women – 52·36% | Rate of PPH | 2011-2017 |
| **Ma et al.^63^** | 1996 | Australia | Cohort study | To examine the obstetric profiles and pregnancy outcomes of immigrant women in New South Wales | 258294 | Australian – 73·31%  Europe – 7·76%  Asia – 7·53%  Middle East – 3·94%  America – 1·30%  NZ/Oceania – 3·69%  Africa – 0·91% | Maternal demographics, obstetric outcomes including pregnancy and postpartum complications | 1990-1992 |
| **MacDorman et al.^64^** | 2021 | USA | Observational study | To better understand racial and ethnic disparities in US maternal mortality. | 615 | Non-Hispanic White – 37·40%  Non-Hispanic Black – 37·72%  Hispanic – 17·72% | Maternal mortality rate and cause of death | 2016-2017 |
| **MacMullen et al.^65^** | 2006 | USA | Retrospective cohort study | To examine the relationship between race and adverse  maternal outcomes in women with asthma | 13990 | White – 63·67%  African American – 24·09%  Hispanic – 10·25%  Asian/Pacific Islander – 1·99% | 11 adverse maternal outcomes across racial groups | 1998-1999 |
| **Maeland et al.^66^** | 2019 | Norway | Retrospective cohort study | To assess the pregnancy outcome of low-risk pregnancies for women originating from non-Western  countries compared with ethnic Norwegian women. | 9392 | Ethnic Norweigan – 78·83%  East Europe – 5·31%  Middle East – 1·47%  . America – 0·91%  Asia – 4·67%  Africa – 2·39%  Western – 10·42% | Relative risk of emergency caesarean section or postpartum haemorrhage by country  of origin | 2009-2015 |
| **Main et al.^67^** | 2020 | USA | Cross-sectional study | To evaluate the impact of a haemorrhage quality-improvement collaborative on racial disparities in severe maternal morbidity from haemorrhage | 1292718 | White – 32·7%  Black – 5·9%  Asian – 13·7%  Hispanic – 42·9%  Other – 4·8% | Severe maternal morbidity rates in women with obstetric haemorrhage | 2011-2016 |
| **Marchant et al.^68^** | 2006 | UK | Case-control study | To determine risk factors for excessive and/  or prolonged vaginal bleeding and  uterine infection from 24 h to 3 months postpartum | 729 | Social class based on occupation of woman  Unemployed – 4·53%  Class 1 & 2 – 15·36% Class 3 NM – 14·13% Class 3M (used as reference) – 12·76%  Class 3&4 – 9·05%  Housewife – 32·65% | ICD codes for delayed and secondary PPH and puerperal sepsis | 1994-1995 |
| **Mone et al.^69^** | 2015 | UK | Retrospective cohort study | To observe the incidence of antenatal risk-factors and adverse maternal outcome in  women of East Timorese origin within a UK population | 7252 | East Timorese – 0·58%  Standard UK maternity population – 99·42% | Patient demographic factors, antenatal, intrapartum and postnatal risk factors and complications | 2011-2012 |
| **Naess-Andresen et al.^70^** | 2022 | Norway | Cohort study | To assess the prevalence of  anaemia and iron deficiency 14 weeks postpartum, and associations with ethnicity and  clinical factors in a multi-ethnic population. | 573 | Western Europe – 37·87% East Europe – 5·93%  Asia – 33·16%  Sub-saharan Africa – 6·63%  Middle East – 16·40% | Postpartum anaemia and iron deficiency | 2008-2010 |
| **Nyflot et al.^71^** | 2017 | Norway | Case-control study | To evaluate risk  factors for severe PPH, taking into consideration prepregnancy,  antenatal and intrapartum variables | 3123 | Europe/USA/Oceania – 80·69%  Middle East/. Africa – 5·51%  Latin-America – 1·15%  Asia – 8·01%  Sub-Saharan Africa – 4·64% | Estimated blood loss > 1500mls or the need for blood transfusion for severe bleeding | 2008-2011 |
| **Oteng-Ntim et al.^72^** | 2013 | UK | Cross-sectional study | To quantify the proportion of adverse pregnancy outcome attributable to maternal obesity | 23668 | White – 54%  Black – 34%  Asian – 5% Oriental – 3%  Other – 4% | Maternal outcomes: diabetes, type of delivery, post-partum haemorrhage, and preterm  delivery. Perinatal outcomes: macrosomia, low birth weight, admission to neonatal intensive care/special care baby unit,  and perinatal death. | 2004-2008 |
| **Pacquier et al.^73^** | 2020 | Belgium | Retrospective cohort study | To assess whether perinatal complications were similar to recent immigrants to those in long term residents | 892 | Recent immigrants – 25·78%  Long-term residents – 74·22% | Sociodemographic data, baseline health status,  prenatal care, obstetrical and neonatal complications | 2016-2017 |
| **Prick et al.^74^** | 2015 | Netherlands | Retrospective cohort study | To assess regional variation in severe PPH in the Netherlands | 1599867 | Western – 84·5%  Non-Western – 15·5% | Incidence of severe PPH (defined as blood loss > 1000mls) | 2000-2008 |
| **Reime et al.^75^** | 2012 | Germany | Retrospective cohort study | To examine the association between region of origin and severe illness  bringing a mother close to death (near-miss). | 441199 | Germany – 83·7%  Western countries – 1·4%  Mediterranean – 2·2%  Central & East Europe – 5·5%  Middle East – 5·0%  Asia – 0·9%  African/Latin America/Other – 1·3% | Hysterectomy, haemorrhage,  eclampsia and sepsis rates | 2001-2007 |
| **Ruiter et al.^76^** | 2019 | Netherlands | Retrospective cohort study | To study the incidence of PPH and MROP and their recurrence rate in a subsequent pregnancy | 359737 | Non-Caucasian ethnicity – 11·46%  Caucasian – 88·54% | Postpartum haemorrhage (defined as >1000 mL after vaginal delivery or Caesarean section) and manual removal of the placenta | 1999-2009 |
| **Salim et al.^77^** | 2012 | Israel | Retrospective case-control study | To compare intrapartum outcomes between ethnic Ethiopian women and the general obstetric population  in Israel. | 1728 | Ethiopian - 33·33% Israeli White women – 66·67% | Incidence of operative delivery | 2004-2011 |
| **Shen et al.^78^** | 2005 | USA | Retrospective cohort study | To examine racial disparities in ad-  verse maternal outcomes among four ethnic groups in the United States. | 1030350 | White – 62·42% African American – 15·70%  Hispanic – 17·85%  Asian – 4·02% | Maternal outcomes including: preterm labour, hypertensive  disorders of pregnancy, gestational diabetes,  antepartum haemorrhage, membrane disorders,  caesarean section, and postpartum haemorrhage. | 1998-1999 |
| **Siddiqui et al.^79^** | 2017 | USA | Retrospective cohort study | To test the hypothesis that higher risk of mortality seen in Asian American and Pacific Islander women id explained by differences in demographics and comorbidities compared to White women | 21,898,501 | Caucasian -91·16%  Asian/Pacific Islander – 8·84% | Inpatient mortality rate, and the presence of severe maternal morbidities | 2002-2013 |
| **Snowden et al.^80^** | 2020 | USA | Before and after study | To test whether Minnesota's blended payment policy had differential effects  on caesarean use and maternal morbidity among black women and white women  in Minnesota, as compared to six control states | 426998 | Black – 31·27%  White – 68·73% | Primary outcome was caesarean birth, and secondary outcomes  were maternal morbidity (composite), postpartum haemorrhage, and chorioamnionitis | 2006-2012 |
| **Staniczenko et al.^81^** | 2018 | USA | Cross-sectional study | To increase knowledge on trends in and implications of unstable housing during pregnancy | 73081805 | Stable housing – 99·97%  Unstable housing – 0·03% | Adverse pregnancy outcomes  including antepartum haemorrhage, preterm delivery, caesarean delivery, hypertensive disorders of pregnancy,  postpartum haemorrhage, and CDC severe maternal morbidity excluding blood transfusion. | 2000-2018 |
| **Stephenson et al.^82^** | 2015 | USA and Canada | Cohort study (secondary analysis of MVI trial) | To compare labour outcomes across race/ethnicity in women undergoing prostaglandin labour  induction. | 1195 | White – 49·12%  Hispanic – 30·38%  Black – 20·50% | Time to delivery, adverse maternal outcomes such as PPH, uterine contractile abnormalities, side effects to medication, neonatal outcomes | 2006-2007 |
| **Taylor et al.^83^** | 2022 | USA | Retrospective cohort study | To determine prevalence, risk factors, and causes for PPH in our obstetric population in  South-Central Louisiana | 30674 | White/Caucasian – 51·5%  Black/African American – 37·5%  Other – 10·9% | Incidence of PPH (defined as blood loss > 1000mls within 24hrs of birth) | 2015-2020 |
| **Tucker et al.^84^** | 2007 | USA | Observational study | To determine whether differences in the prevalences of  5 specific pregnancy complications  explained the disproportionate risk of pregnancy-related mortality  for Black women compared with White women in the United States | Not stated | Black  White  (percentage of study population in each group not stated in paper) | Prevalence rate and case-fatality rate for Preeclampsia, eclampsia, abruption, placenta praevia, PPH | 1988-1999 |
| **Urquia et al.^85^** | 2017 | Canada | Retrospective cohort study | To quantify the risk of severe maternal  morbidity (SMM) according to maternal country of birth in Canada | 1252543 | Immigrant women – 26·79%  Canadian-born women – 73·21% | Composite  indicator of SMM used for surveillance. | 2002-2012 |
| **Versi et al.^86^** | 1995 | UK | Retrospective cohort study | To study the obstetric outcome of women of Bangladeshi origin in comparison with the white Caucasian population in East London | 16718 | Bangladeshi women – 38·64%  White Caucasian women – 45·41% | Maternal: antenatal complications induction and augmentation rates,  analgesia and anaesthesia, instrumental delivery and caesarean section rates, third stage complications. Fetal: gestation and weight at birth; perinatal mortality rates. | 1987-1991 |
| **Vinograd at al.^87^** | 2015 | Israel | Retrospective cohort study | To determine the effect of a history of  placenta accreta on subsequent pregnancies. | 239640 | Jews  Bedoins  (percentage of study population in each group not stated in paper) | Placenta accreta, pregnancy outcomes including anaemia, wound infection, PPH, postpartum fever, infection | 1988-2010 |
| **Von Katterfeld et al.^88^** | 2011 | Australia | Cohort study | To examine the obstetric profiles of foreign-born women in WA using routinely collected perinatal data | 208982 | Australia – 71·65%  Oceania – 4·06%  NW Europe – 10·83%  SE Europe – 1·67%  N African & Middle East – 1·10%  Sub-Saharan Africa – 2·27%  Asia – 7·22%  Americas – 1·19% | Complications of pregnancy labour and delivery (including PPH) and obstetric interventions | 1998-2006 |
| **Waterstone et al.^89^** | 2001 | UK | Case-control study | To estimate the incidence and predictors of  severe obstetric morbidity. | 2938 | White – 82·85%  Black – 12·29%  Other – 4·87% | Severe obstetric morbidity including haemorrhage and preeclampsia | 1997-1998 |
| **Westerway et al.^90^** | 2003 | Australia | Cohort study | To compare rates of fetal macrosomia (birthweight >4000 g) and birth complications in both Chinese  women immigrants and Caucasian women for two time periods: 1992 and 1999–2000 | 4497 | Caucasian – 90·35%  Chinese – 9·65% | Fetal macrosomia, obstetric intervention and PPH (defined as EBL > 500mls) | 1992 and 1999-2000 |
| **Yeshitila et al.^91^** | 2024 | Australia | Cross-sectional study | To examine trend and disparities in adverse perinatal outcomes among women of refugee background using population-based data | 754270 | Refugee background – 4·35%  Australian born – 95·65% | Maternal outcomes: perineal tears, postpartum haemorrhage, puerperal sepsis, abnormal labour, postnatal depression, admission to intensive care. Neonatal outcomes: preterm birth, admission to neonatal intensive care, small for gestational age, APGAR < 7 at 5 mins, stillbirth, neonatal death | 2003-2017 |
| **Zwart et al.^92^** | 2010 | Netherlands | Cohort study | To assess ethnic disparity in outcomes for women that experienced severe acute maternal morbidity | 358874 | Non- Western immigrants – 21·1%  Western women -78·9% | Severe acute maternal morbidity including ICU admission, uterine rupture, eclampsia, massive obstetric haemorrhage | 2004-2006 |

Table S1: Baseline Characteristics of included Studies in the systematic review of associations between ethnicity, socioeconomic status and postpartum haemorrhage

**Table S2: Study Quality Assessment for Cohort Studies**

| Study ID | Representative of the Exposed Cohort | Selection of the Non-exposed cohort | Ascertainment of exposure | Demonstration that the Outcome of interest was not present at the start of the study | Comparability of Cohort | Assessment of outcome | Follow up duration to capture outcomes | Adequacy of follow up | Total Score |
| --- | --- | --- | --- | --- | --- | --- | --- | --- | --- |
| Al-Zirqi 2008 | * | * | * | * | * | * | * | * | 8 |
| Bakken 2015 | * | * | * | * | * | * | * |  | 7 |
| Bakken 2017 |  | * | * | * | * | * | * |  | 6 |
| Biguzzi 2012 |  | * | * | * | * | * | * | * | 7 |
| Briley 2014 | * | * | * | * | * | * | * |  | 7 |
| Bryant 2012 | * | * | * | * | * | * | * | * | 8 |
| Buckley 2022 |  | * | * | * | * | * | * | * | 7 |
| Butwick 2014 |  | * | * | * | * | * | * | * | 7 |
| Cabacungan 2012 | * | * | * | * | * | * | * | * | 8 |
| Calje 2023 |  | * | * |  | * | * | * |  | 5 |
| Chalouhi 2015 |  | * | * | * |  | * | * |  | 5 |
| Chichakli 1999 | * | * | * |  |  | * | * | * | 6 |
| Cho 2022 | * | * | * | * | * | * | * | * | 8 |
| Creanga 2012 |  |  | * |  |  | * | * | * | 4 |
| Creanga 2014 | * | * | * | * | * | * | * | * | 8 |
| Davey 2020 | * | * | * | * | * | * | * | * | 8 |
| Davidson 2022 | * | * | * | * |  | * | * | * | 7 |
| Davis 2012 |  | * | * | * | * | * | * |  | 6 |
| Drassinower 2014 |  | * | * | * | * | * | * | * | 7 |
| El-Sayad 2020 |  | * | * | * | * |  | * | * | 6 |
| Erickson 2020 | * | * | * | * | * | * | * | * | 8 |
| Eslier 2020 | * | * | * | * | * | * | * |  | 7 |
| Estrada 2022 |  | * | * | * | * | * | * | * | 7 |
| Fadel 2019 |  |  | * |  |  | * | * |  | 3 |
| Faulks 2023 | * | * | * | * | * | * | * | * | 8 |
| Flores 2015 |  | * | * | * | * | * | * | * | 7 |
| Ford 2007 | * | * | * | * | * | * | * | * | 8 |
| Fotovati 2024 |  | * | * | * | * | * | * |  | 6 |
| Fuller 2023 |  | * | * | * |  | * | * |  | 5 |
| Fyfe 2012 |  | * | * | * | * | * | * | * | 7 |
| Girault 2018 |  | * | * | * | * |  | * |  | 5 |
| Grob 2022 | * | * | * | * |  | * | * | * | 7 |
| Grobman 2015 | * | * | * | * | * | * | * | * | 8 |
| Guendelman 2006 | * | * | * | * | * | * | * | * | 8 |
| Guomundsdottir 2021 | * | * | * | * | * | * | * | * | 8 |
| Gyamfi-Bannerman 2018 | * | * | * | * | * | * | * | * | 8 |
| Halloran 2012 |  | * | * | * | * | * | * | * | 7 |
| Hamilton 2021 | * | * | * | * |  | * | * |  | 6 |
| Hartenbach 2020 | * | * | * |  | * | * | * | * | 7 |
| Harvey 2017 | * | * | * | * | * | * | * | * | 8 |
| Jardine 2022 | * | * | * | * | * | * | * | * | 8 |
| Katz 2022 | * | * | * | * | * | * | * | * | 8 |
| Lawson 2020 | * | * | * | * | * | * | * | * | 8 |
| Lucas 2021 |  | * | * | * | * | * | * |  | 6 |
| Ma 1996 | * | * | * | * |  | * | * | * | 7 |
| MacDorman 2021 | * | * | * |  |  | * | * |  | 5 |
| Macmullen 2006 |  | * | * | * | * | * | * | * | 7 |
| Maeland 2019 | * | * | * | * | * | * | * | * | 8 |
| Mone 2015 | * | * | * | * |  | * | * | * | 7 |
| Naess-Andresen 2022 | * | * | * | * | * | * | * |  | 7 |
| Paquier 2020 | * | * | * |  | * |  | * | * | 6 |
| Prick 2015 | * | * | * | * | * | * | * | * | 8 |
| Reime 2012 | * | * | * | * | * | * | * |  | 7 |
| Ruiter 2019 | * | * | * | * | * | * | * |  | 7 |
| Shen 2005 | * | * | * | * |  | * | * | * | 7 |
| Siddiqui 2017 | * | * | * |  | * | * | * | * | 7 |
| Snowden 2020 | * |  | * | * | * | * | * |  | 6 |
| Stephenson 2015 |  | * | * | * | * | * | * | * | 7 |
| Taylor 2022 | * | * | * | * | * | * | * | * | 8 |
| Tucker 2007 | * |  | * |  |  | * | * |  | 4 |
| Urquia 2017 | * | * | * | * | * | * | * | * | 8 |
| Versi 1995 |  | * |  | * |  |  | * |  | 3 |
| Vinograd 2015 |  | * | * | * | * | * | * |  | 6 |
| von Katterfeld 2011 | * | * | * | * |  | * | * | * | 7 |
| Westerway 2003 |  | * | * | * | * | * | * |  | 6 |
| Zwart 2010 | * | * | * | * | * | * | * | * | 8 |

Table S2: Quality assessment overview of cohort studies included in the systematic review of associations between ethnicity, socioeconomic status and postpartum haemorrhage. N =66

**Table S 3: Study Quality Assessment for Case-Control Studies**

| Study ID | Case Definition adequate | Representativeness of cases | Selection of controls | Definition of controls | Comparability of cases and controls on the basis of design or analysis | Ascertainment of exposure | Same method of ascertainment for cases and controls | Non-response rate | Total Score |
| --- | --- | --- | --- | --- | --- | --- | --- | --- | --- |
| Elkington 2023 |  | * |  | * | * |  | * |  | 4 |
| Kanthasamy 2013 |  | * |  | * |  | * | * | * | 5 |
| Marchant 2006 | * | * | * | * | * | * | * |  | 7 |
| Nyflot 2017 | * | * |  | * | * | * |  |  | 5 |
| Salim 2012 |  | * | * | * |  | * | * |  | 5 |
| Waterstone 2001 |  | * | * | * | * | * |  |  | 5 |

Table S3: Quality assessment overview of case-control studies included in the systematic review of associations between ethnicity, socioeconomic status and postpartum haemorrhage. N =6

**Table S4: Study Quality Assessment for Cross-Sectional Studies**

| **Study ID** | **Representativeness of the Sample** | **Sample Size** | **Non-included Subjects** | **Study Controls for Confounding Factors** | **Study Controls for Other Factors** | **Assessment of the Outcome** | **Statistical Test** | **Total Score** |
| --- | --- | --- | --- | --- | --- | --- | --- | --- |
| Almeida 2014 | * | * |  |  |  | * | * | 4 |
| Brown 2021 | * | * | * | * |  | * | * | 6 |
| Guglielminotti 2022 |  | * |  | * |  | * | * | 4 |
| Main 2020 | * | * | * | * |  | * | * | 6 |
| Oteng-Ntim 2013 | * | * |  | * |  | * | * | 5 |
| Staniczenko 2018 | * | * |  |  |  | * |  | 3 |
| Yeshitila 2024 | * | * |  | * |  | * | * | 5 |

Table S4: Quality assessment overview of cross-sectional studies included in the systematic review of associations between ethnicity, socioeconomic status and postpartum haemorrhage. N =7

**Funnel Plots Assessing for Publication Bias**


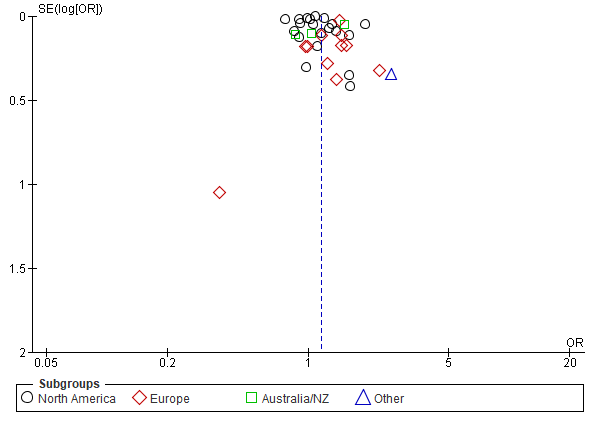


Figure S1: Funnel plot of the unadjusted odd ratio (on the log scale) versus standard error of the log odds ratio for the included studies in the Black vs. White analysis.


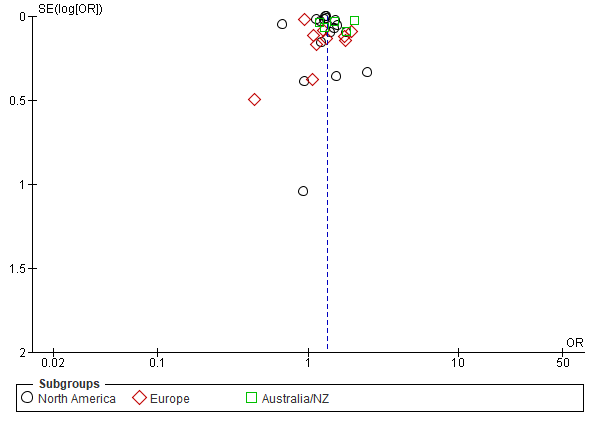


Figure S2: Funnel plot of the unadjusted odd ratio (on the log scale) versus standard error of the log odds ratio for the included studies in the White vs. Asian analysis.


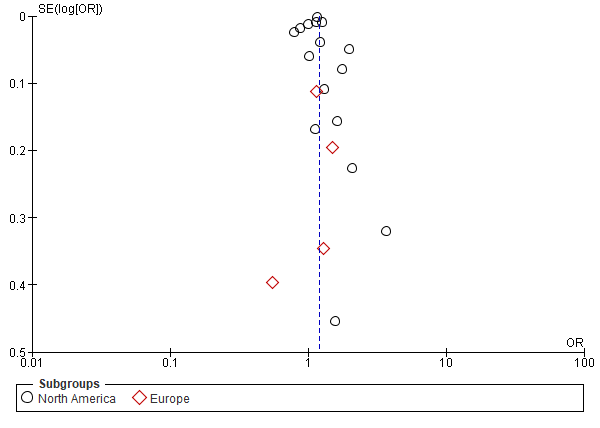


Figure S3: Funnel plot of the unadjusted odd ratio (on the log scale) versus standard error of the log odds ratio for the included studies in the White vs. Hispanic analysis.

**
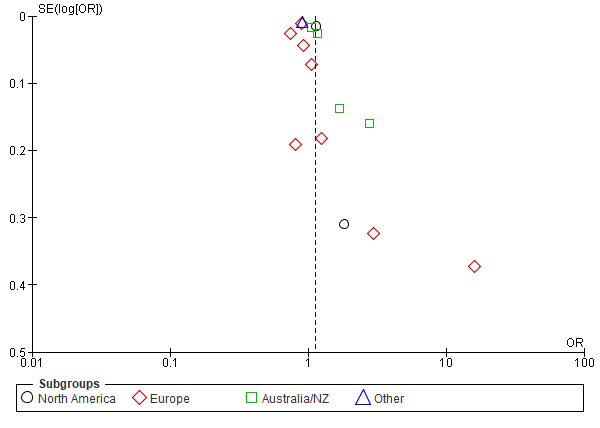
**

Figure S4: Funnel plot of the unadjusted odd ratio (on the log scale) versus standard error of the log odds ratio for the included studies in the majority ethnic group vs minority ethnic group analysis.

**Table S5: Subgroup Analysis by Study Continent**

|  | North America | Europe | Australia/New Zealand | Other |
| --- | --- | --- | --- | --- |
| White vs. Black | 1·11 (1·03,1·19)  N= 20  *I*^2^ = 97% | 1·33 (1·19, 1·49)  N = 11  *I*^2^ = 35% | 1·12 (0·78, 1·60)  N = 3  *I*^2^ = 93% | 2·59 (1·30, 5·13)  N= 1  *I*^2^ = N/A |
| White vs. Asian | 1·27 (1·22, 1·33)  N = 17  *I*^2^ = 95% | 1·27 (1·01, 1·60)  N = 10  *I*^2^  = 92% | 1·51 (1·20, 1·91)  N = 5  *I^2^ =* 98% | ·· |
| White vs. Hispanic | 1·21 (1·12, 1·29)  N = 16  *I*^2^ = 98% | 1·16 (0·87, 1·56)  N = 4  *I*^2^ = 43% | ·· | ·· |
| Majority vs. Minority | 1·30 (0·86, 1·97)  N = 2  *I*^2^ = 57% | 1·07 (0·91, 1·24)  N = 8  *I*^2^ = 95% | 1·38 (1·16, 1·63)  N = 4  *I*^2^ = 94% | 0·91 (0·89, 0·92)  N = 1  *I*^2^ = N/A |

Table S5: Subgroup analysis with regards to study continent. Values are represented as odds ratio (95% confidence interval). N = number of studies. *I^2^* statistic as a measure of heterogeneity.

**Figure S5: Forest plot for risk of PPH for Asian Vs White ethnicity**

**
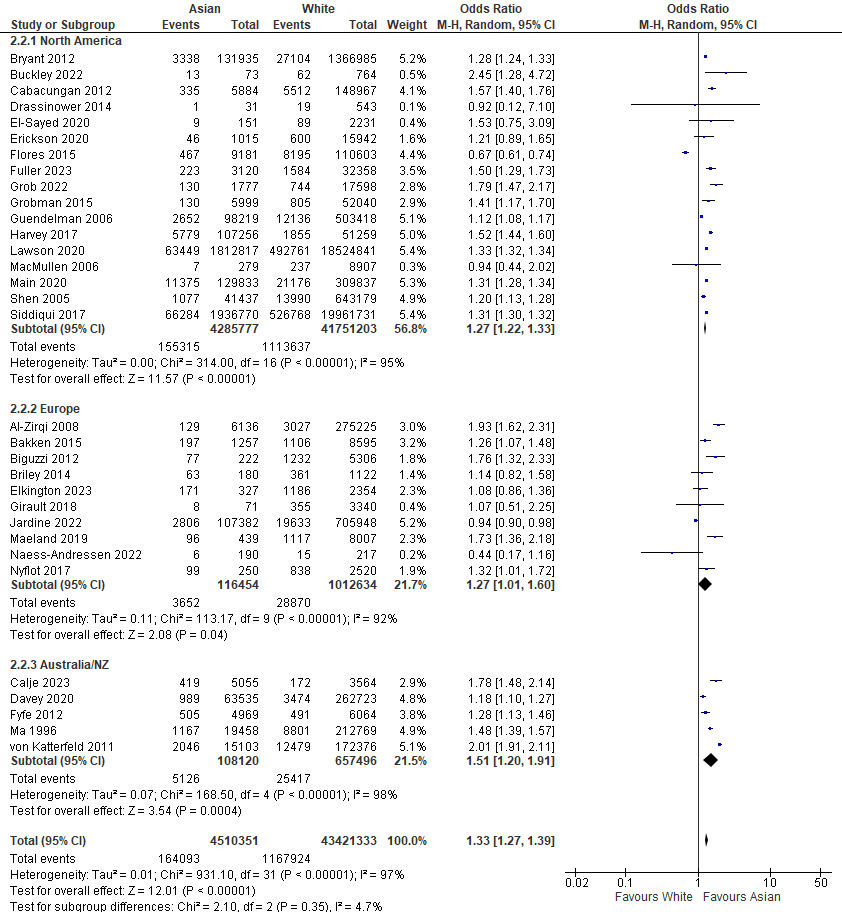
**

Figure S5: Risk of postpartum haemorrhage in White versus Asian ethnicity, stratified by study continent. CI indicates confidence interval.

**Figure S6: Forest plot for risk of PPH for Hispanic vs White ethnicity**

**
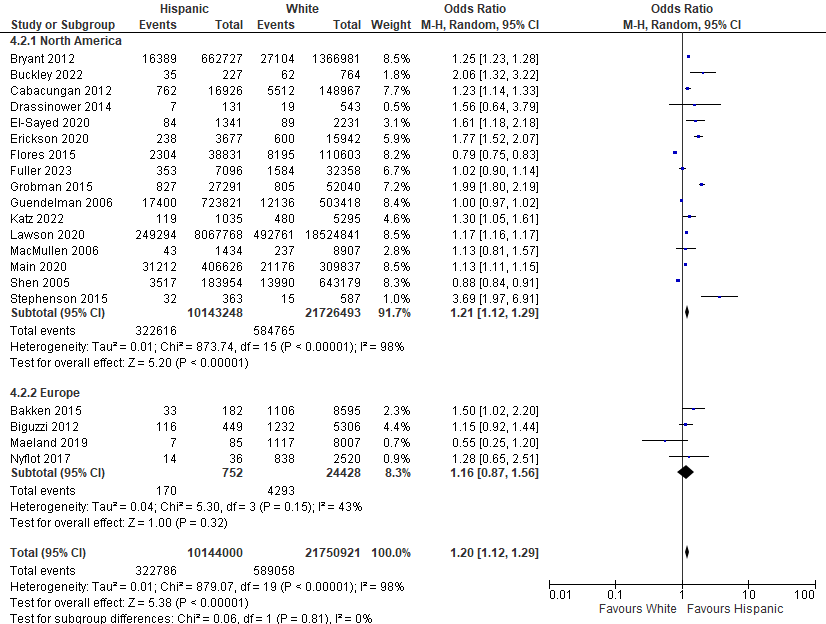
** Figure S6: Risk of postpartum haemorrhage in White versus Hispanic ethnicity, stratified by study continent. CI indicates confidence interval.

**Figure S7: Forest plot for risk of PPH for Majority vs Minority ethnic groups**

**
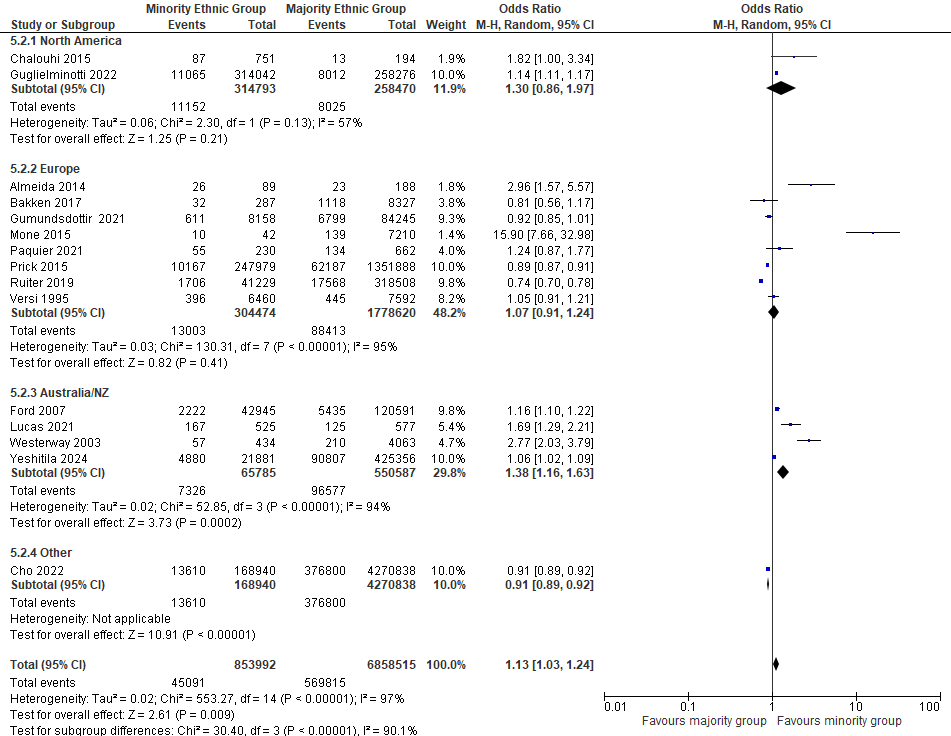
** Figure S7: Risk of postpartum haemorrhage in majority ethnic group vs. minority ethnic group within the study, stratified by study continent. CI indicates confidence interval.

**Table S6: Subgroup Analysis by Study Continent and Time Period**

| **White vs. Black** | 1990–1999 | 2000–2009 | 2010–2019 |
| --- | --- | --- | --- |
| North America | 0·95 (0·86, 1·07)  N = 3  *I*^2^ = 89% | 1·09 (0·97, 1·24)  N = 8  *I*^2^ = 98% | 1·22 (1·13, 1·31)  N = 8  *I*^2^ = 57% |
| Europe | 0·97 (0·68, 1·39)  N = 1  *I*^2^ = N/A | 1·39 (1·18, 1·64)  N = 7  *I*^2^ = 22% | 1·23 (0·94, 1·61)  N = 4  *I*^2^ = 50% |
| Australia/NZ | 1·26 (0·88, 1·82)  N = 2  *I*^2^ = 91% | 0·86 (0·70, 1·07)  N = 1  *I*^2^ = N/A | ·· |
| Other | ·· | 2·59 (1·30, 5·13  N = 1  *I*^2^ = N/A | ·· |
| **White vs. Asian** |  |  |  |
| North America | 1·25 (1·04, 1·50)  N = 4  *I*^2^ = 96% | 1·22 (1·16, 1·29)  N =8  *I*^2^ = 97% | 1·54 (1·29, 1·82)  N = 5  *I*^2^ = 76% |
| Europe | 1·93 (1·62, 2·31)  N = 1  *I*^2^ = N/A | 1·36 (1·12, 1·66)  N = 6  *I*^2^ = 65% | 0·94 (0·91, 0·98)  N = 3  *I*^2^ = 0% |
| Australia/NZ | 1·72 (1·28, 2·33)  N = 2  *I*^2^ = 98% | 1·21 (1·12, 1·30)  N = 2  *I*^2^ = 20% | 1·78 (1·48, 2·14)  N = 1  *I*^2^ = N/A |
| Other | ·· | ·· | ·· |
| **White vs. Hispanic** |  |  |  |
| North America | 0·95 (0·85, 1·07)  N = 3  *I*^2^ = 94% | 1·32 (1·18, 1·48)  N = 8  *I*^2^ = 98% | 1·24 (1·08, 1·43)  N =5  *I*^2^ = 76% |
| Europe | ·· | 1·16 (0·87, 1·56)  N = 4  *I*^2^ = 43% | ·· |
| Australia/NZ | ·· | ·· | ·· |
| Other | ·· | ·· | ·· |
| **Majority vs. Minority** |  |  |  |
| North America | ·· | 1·82 (1·00, 3·34)  N = 1  *I*^2^ = N/A | 1·14 (1·11, 1·17)  N = 1  *I*^2^ = N/A |
| Europe | 0·89 (0·72, 1·09)  N = 3  *I*^2^ = 94% | 0·89 (0·87, 0·91)  N = 2  *I*^2^ = 0% | 3·78 (0·87, 16·33)  N = 3  *I*^2^ = 95% |
| Australia/NZ | 1.77 (0.75, 4.16)  N = 2  *I*^2^ = 97% | 1.06 (1.02,1.09)  N = 1  *I*^2^ = N/A | 1.69 (1.29, 2.21)  N = 1  *I*^2^ = N/A |
| Other | ·· | 0·91 (0·89, 0·92)  N = 1  *I*^2^ = N/A | ·· |

Table S6. Subgroup analysis with regards to study continent over time. Values are represented as odds ratio (95% confidence interval). N = number of studies. I^2^ statistic as a measure of heterogeneity

**Table S7: Severe Maternal Morbidity from Haemorrhage in Different Ethnic Groups**

|  | OR (95% CI) for SMM from Haemorrhage |
| --- | --- |
| White Vs. Black | 1·57 (1·25, 1·98)  N = 11  *I^2^* = 99% |
| White Vs. Asian | 1·39 (1·25, 1·54)  N = 12  *I^2^* = 97% |
| White Vs. Hispanic | 1·30 (1·18, 1·42)  N = 9  *I^2^* = 96% |

Table S7. Pooled effect estimates for SMM from Haemorrhage for Black, Asian and Hispanic ethnic groups. Values are represented as odds ratio (95% confidence interval). N = number of studies. I^2^ statistic as a measure of heterogeneity

**Figure S8: Pooled Effect Estimate for Maternal Mortality from Haemorrhage**

**
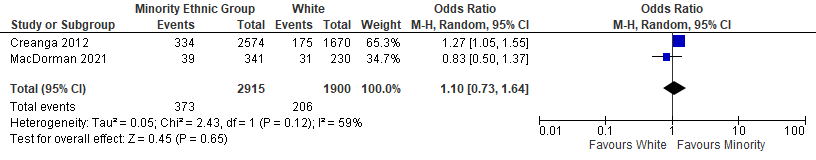
**

Figure S8. Risk of maternal mortality from haemorrhage for minority ethnic group versus white women. CI indicates confidence interval.
